# Variable immunodeficiency score upfront analytical link (VISUAL), a proposal for combined prognostic score at diagnosis of common variable immunodeficiency

**DOI:** 10.1038/s41598-021-91791-2

**Published:** 2021-06-09

**Authors:** Kissy Guevara-Hoyer, Adolfo Jiménez-Huete, Julia Vasconcelos, Esmeralda Neves, Silvia Sánchez-Ramón

**Affiliations:** 1 Department of Immunology, IML and IdSSC, Hospital Clínico San Carlos, Madrid, Spain; 2 Department of Immunology, Ophthalmology and ENT, School of Medicine, Complutense University, Madrid, Spain; 3 Immunodeficiency Interdepartmental Group (GIID), Madrid, Spain; 4 Department of Neurology, Hospital Ruber Internacional, Madrid, Spain; 5 Department of Immunology, Centro Hospitalar e Universitário do Porto, Porto, Portugal

**Keywords:** Immunology, Biomarkers, Medical research

## Abstract

The broad and heterogeneous clinical spectrum that characterizes common variable immunodeficiency (CVID) is associated with quite different disease course and prognosis, highlighting the need to develop tools that predict complications. We developed a multianalyte VISUAL score (variable immunodeficiency score upfront analytical link) aimed to predict severity using individual CVID patient data at baseline of a cohort of 50 CVID patients from two different centers in Portugal and Spain. We retrospectively applied VISUAL to the CVID clinical severity scores proposed by Ameratunga and Grimbacher after 15 years follow-up of our cohort. VISUAL score at CVID diagnosis showed adequate performance for predicting infectious and non-infectious severe complications (Cluster B). Compared to switched memory B lymphocyte phenotype alone, VISUAL provided a more accurate identification of clinically meaningful outcome, with significantly higher sensitivity (85% *vs* 55%, p = 0.01), and negative predictive value (77% *vs* 58%) and AUC of the ROC curves (0.72 *vs* 0.64), with optimal cut-off level of 10. For every increase of 1 point in the VISUAL scale, the odds of being in the higher risk category (Cluster B) increased in 1.3 (p = 0.005) for Ameratunga’s severity score and 1.26 (p = 0.004) for Grimbacher’s severity score. At diagnosis of CVID, VISUAL score ≥ 10 showed 8.94-fold higher odds of severe prognosis than below this threshold. Kaplan–Meier estimates for the VISUAL ≥ 10 points showed significantly earlier progression to Cluster B than those with VISUAL < 10 (*p* = 0.0002). This prognostic laboratory score might allow close monitoring and more aggressive treatment in patients with scores ≥ 10 on a personalized basis approach. Further studies are needed to prospectively validate VISUAL score.

## Introduction

Common variable immunodeficiency (CVID) is the most commonly symptomatic primary immunodeficiency disorders (PIDD)^1^, with a prevalence of 1 in 25,000 to 50,000 individuals. Both genders are equally affected^2,3^. The broad and heterogeneous clinical spectrum that characterizes CVID is associated with quite different disease course and prognosis, highlighting the need of prediction tools for complications. Combination of biomarkers in scores may assist in providing a more accurate picture of the disease burden in the individual patient. Although these tools are essentially used for research purposes, very few are used in the clinical routine for CVID patient populations. The EUROclass trial was designed to define CVID subgroups according to memory B lymphocyte phenotype, concealing the previous classification schemes of Paris and Freiburg^4^. This classification resulted predictive for autoimmune disorders, lymphoproliferative or granulomatous complications, but did not encompass the extent of manifestations associated with the severity of disease. World CVID experts have proposed that CVID diagnosis should be based on clinical manifestations and laboratory criteria, and then complemented by new biomarkers such as B and T lymphocyte phenotype, lymphoproliferative studies, activation markers and genetic tests for a more precise diagnosis^5–9^. The clinical phenotyping classification proposed by Chapel^7^, the scoring system to initiate immunoglobulin replacement therapy (IgRT) proposed by Cunningham-Rundles^10^, and the clinical severity scores proposed by Ameratunga^11^ and Grimbacher^12^ are widely used in clinical practice.

Here we sought to determine the usefulness of a combined multianalyte CVID prognostic score at diagnosis, namely VISUAL (variable immunodeficiency score by upfront analytical link), to predict prognosis in CVID patients.

## Methods

We conducted an observational study to assess the clinical performance of a prognostic VISUAL score retrospectively applied on 50 CVID patients followed at the Departments of Clinical Immunology of the Centro Hospitalar do Porto, Portugal and the Hospital Clínico San Carlos, Madrid, Spain. The study was approved by the Ethics committee of both centers.

### Patients

Inclusion criteria of CVID diagnosis included a reduction of at least two serum immunoglobulin isotypes (IgG, IgM or IgA) by two standard deviations (< 2SD) from normal mean values for age (> 4 years), evidence of specific antibodies deficiency, and exclusion of other causes of hypogammaglobulinemia, according to the classification of European Society of Immune Deficiencies (ESID) and the Pan-American Group for Immune Deficiency (PAGID)^13,14^. The diagnosis of combined immune deficiency was excluded in those patients with suspected CVID and CD4^+^ T-lymphopenia.

### Clinical information

Clinical information was compiled on each CVID subject, including history of any infections, pneumonias; severe infections, chronic lung disease, bronchiectasis, pulmonary granulomata or lymphoid interstitial pneumonia, autoimmune diseases, granulomatous infiltration, presence of splenomegaly, splenectomy, skin and musculoskeletal manifestations, endocrine, cardiac, kidney and liver disorders, as well as allergies, and history of malignancy, among others^11,12^ (Suppl. Table S1).

According to the data collected, all patients had a genetic study performed during their follow-up (pending results of 3 patients). Of the remaining 47 patients, only five patients had a known mutation (10.63%), none of them being current monogenic CVID-like mutations (Suppl. Table S2).

### VISUAL score

We applied the VISUAL score using combined immunological biomarkers at CVID diagnosis retrospectively to our cohort of CVID patients. Data on initial serum immune globulins and antibody responses to immunization against polysaccharide and protein antigens were collected. For serum immune globulins, we used the age-adjusted normal values^15,16^, the values were below 2SD (IgM < 0.04 g/L and IgA < 0.07 g/L, respectively) as criteria for CVID diagnosis and undetectable values by routine techniques in our laboratories. IgM values between 0.4 and 2.3 g/L were considered normal values^17,18^. Regarding antibody responses to immunization: for protein vaccines, we defined adequate response as a fourfold increase in IgG anti-tetanus toxoid antigen above the pre-vaccination concentration or above 0.15 IU/mL^19–22^; for IgG pneumococcal polysaccharide vaccine responses, a threefold increase in anti-PPV titers above the pre-vaccination concentration or above 11 mg/dL, as previously published^23–27^. Data of switched memory B-lymphocytes (smB) (CD19^+^CD27^+^IgD^−^IgM^−^) and CD4^+^ T-lymphocytes proportions and counts determined by multiparametric flow-cytometry as per routine analysis were collected. Normal smB lymphocytes reference ranges were defined between 6 and 29%^28–31^. To classify categories of smB lymphocytes we used the cut-off points between categories of 6% of smB according to lower limit of cut-off defined as normal^28–31^; 2% of smB according to the EUROclass study^4^; and 1% using standard ROC analysis in a single-center study^32^. CD4^+^ T-lymphocytes varies widely in CVID patients with distinctive complications^33^. CD4^+^ T-lymphocytes counts were categorized according to established HIV immunological stages (≥ 500/μL, stage 1; 200–499, stage 2; and < 200, stage 3)^34^. We further divided stage 1 based on the lower and upper cut-off points considered within normality into low (500–699) and high (> 700/UL) ranges (Table 1). In patients with CD4^+^ T lymphopenia < 200/µL, a differential diagnosis was performed, discarding combined immune deficiency.

**Table 1:** VISUAL score of combined analytical biomarkers at diagnosis.

Point value	1	2	3	4
smB lymphocytes (%)	Normal	< 6%	< 2%	< 1%
IgA (g/L)	Normal-2SD	< 2SD	–	< 0.07
IgM (g/L)	–	–	–	> 230
Specific Ab responses	Normal	Altered only polysaccharide or only protein responses	–	Altered polysaccharide and protein responses
CD4^+^ T-lymphocytes (/µL)	700–1500	500–700	200–500	< 200

The VISUAL score was designed based on combined immunological biomarkers at CVID diagnosis retrospectively to our cohort of CVID patients. Only those variables that proved to be statistically significant in the multivariable tests (ANOVA) for severity score were included. smB lymphocytes, IgA, specific Ab responses, CD4^+^ T-lymphocytes, and increase in serum IgM were scored from 1 to 4 according to the magnitude of the alteration.

### Categorization of severity in CVID

To define severity, we used two clinical scoring systems:

- Ameratunga’s severity score for CVID-associated disorders. This system categorizes different CVID-associated complications according to specific organs and systems and assigns a severity score that ranges from 1 point for mild complications, 5 points for moderate, up to 10 points for severe complications^11^.
- Grimbacher’s severity score for CVID patients evaluates 15 complications with a point score including autoimmunity, lymphoproliferative disorders, CVID enteropathy, malignancy, development of bronchopulmonary pathologies, need for surgical intervention, such as lung surgery or splenectomy and history of severe infections such as chronic sinusitis, meningitis, encephalitis or pneumonia. This scoring system ranges from 0 points for the absence of manifestations to 3 points for the confirmation of the complication, a severe stage or the need for surgical intervention^12^.

### Statistical analysis

The statistical analyses were performed with the R software (3.6.2)^35^ and the packages OptimalCutpoints^36^, boot^37^ and pROC^38^. The diagnostic performance of the VISUAL score was tested against both the Ameratunga’s and Grimbacher’s severity scores. According to the distribution of the severity scores in our series, we decided to categorize this variable into two clusters (Cluster A and Cluster B). The optimal cut-off of the VISUAL score and the sensitivity, specificity and area under the Receiver Operating Characteristic curve (ROC) of the model were calculated with the ROC method implemented in the OptimalCutpoints package^38^. Kaplan–Meier estimates of the cumulative probability of clinical progression to Cluster B were calculated for the two groups, established according to the VISUAL cut-off, and were compared with the log rank test (Mantel-Cox).

In addition, the VISUAL score was compared with the smB lymphocytes using the McNemar test for the sensitivity and specificity, and a bootstrap method for the AUC. A *p* value of 0.05 was considered as statistically significant.

### Ethics

The study was carried out in accordance with relevant guidelines and regulations. The study was approved by the Ethics Committees of both Centro Hospitalar do Porto (076-19 (065-DEFI/066-CE)) and Hospital Clínico San Carlos (19/284-E). Exception of written informed consent of Centro Hospitalar do Porto was approved for the center’s ethics committee, due to the characteristics of the study. Written informed consent of Hospital Clínico San Carlos was obtained from all subject above 18 years in accordance to the Declaration of Helsinki. In subjects under 18 years of age, informed consent was provided by their parents or representatives.

## Results

The cohort comprised 50 CVID patients with gender distribution of female:male of 2:1. The median age at diagnosis was 32 years (range 4–70), and the median evolution of disease was 15.9 years (range 1 to 31) at the present time.

### Development of a multianalyte prognostic score

The VISUAL score was developed from the laboratory parameters at CVID diagnosis of each patient, as follows: (i) a list of candidate variables aimed to predict the clinical severity of CVID patients was analyzed (serum immunoglobulins G, A, M and E, IgG subclasses, production of specific antibodies, B lymphocytes, memory B lymphocyte subsets, CD4^+^ and CD8^+^ T-lymphocytes, natural killer cells, C3 and C4 complement factors); (ii) only those variables that proved to be statistically significant with p values < 0.05 in the multivariable tests (ANOVA) for severity score were included in VISUAL (smB lymphocytes, IgA, specific Ab responses, CD4^+^ T-lymphocytes); (iii) due to the particular clinical significance of the increase in serum IgM described in previous studies^17,39^, IgM was considered within the score. The different analytes of the VISUAL were scored from 1 point for the normal range to 4 points for the absence of smB lymphocytes, IgA, specific Ab responses, CD4^+^ T-lymphocytes, or increase in IgM. Thus, the VISUAL was calculated as the sum of the individual scores ranging from 5 to 20. The resultant multianalyte VISUAL score is shown in the Table 1.

Considering the proportion of smB lymphocytes, we observed that 24% of our patients presented normal levels (> 10%), 36% smB lymphocytes between 6 and 2%; 14% between 2 and 1%, and 26% levels below 1%. Fifty percent of our patients showed undetectable IgA levels, 24% had values < 2SD and 26% presented low to normal levels. Six percent of our cohort presented high serum IgM levels. Regarding antibody responses to immunization, 58% of our patients showed inadequate specific antibodies’ (Ab) responses to both polysaccharide and protein antigens; 28% had only inadequate polysaccharide Ab responses, while 14% only inadequate protein Ab responses. Forty-four percent of our CVID patients presented normal CD4^+^ T-lymphocytes; 12% had CD4^+^ values between 500 and 700 µ/mL, 32% of the cohort between 200 to 500 µ/mL and 12% (6/50) showed levels below 200 µ/mL. These latter 6 patients with CD4^+^ T-lymphocytopenia had received corticosteroids chronically due to clinical manifestations such as moderate asthma, chronic rhinosinusitis, severe GLILD, inflammatory bowel disease, and/or severe pulmonary dysfunction with extensive bronchiectasis (Supp. Table S1).

**Figure 1:**
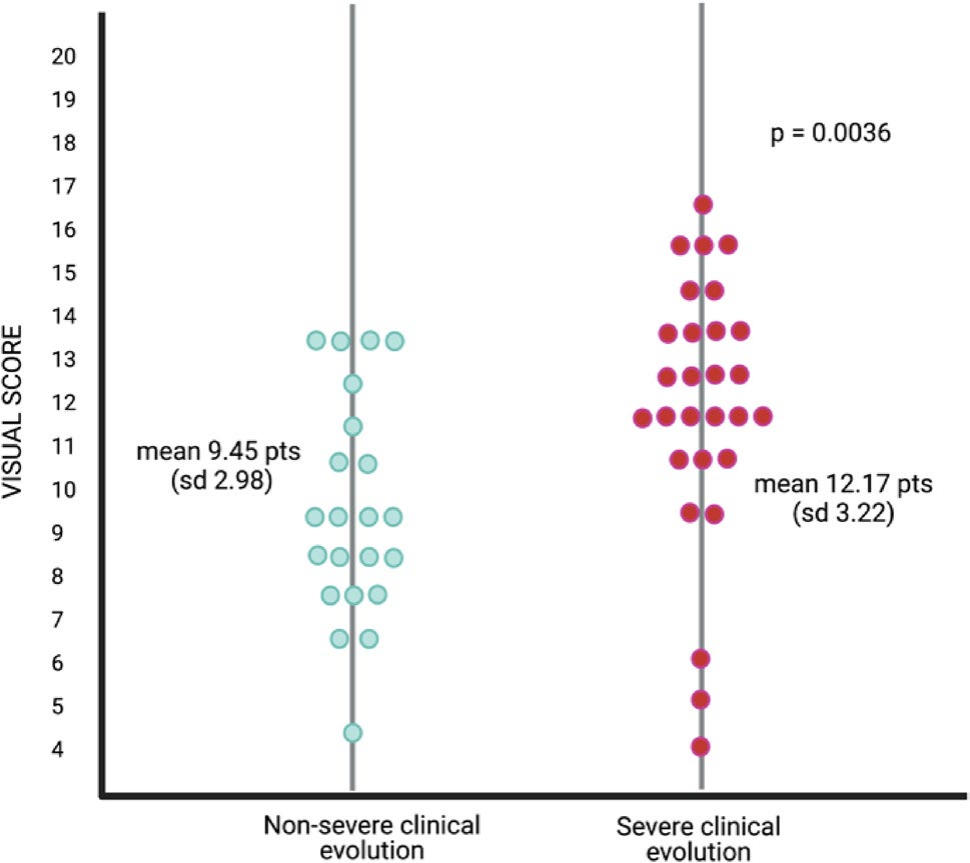
Silhouette plots for the analysis of the two different clusters associated to clinical evolution in our cohort against VISUAL score. Patients with non-severe clinical evolution presented a mean of 9.45 points (SD 2.98) and patients with severe clinical evolution (Cluster B) with a mean of 12.17 points (SD 3.22) (p = 0.0036). Created with BioRender.com.

We analyzed two different clusters associated with clinical evolution in our cohort against VISUAL score: patients with non-severe clinical evolution (Cluster A) presented a mean of 9.45 points (SD 2.98) and patients with severe clinical evolution (Cluster B) with a mean of 12.17 points (SD 3.22) (p = 0.0036) (Fig. 1). When we applied VISUAL distribution to our cohort, a unimodal behavior was observed, with a median of 10 points (Supp. Fig. S1). In order to investigate whether there was an association between VISUAL score and the clinical evolution of our patients based on pre-established clinical scores, we retrospectively applied the Ameratunga’s and the Grimbacher’s severity scores to our cohort (Supp. Fig. S2). In our CVID patients, the Ameratunga’s severity score disclosed a median of 14 points (range 8 to 21 points) and a unimodal behavior^11^. Patients with severe complications or more than one moderate complication amounted to 14 or more score points. The cohort was divided according to the clustering algorithm using 14 points as cut-off into Cluster A (non-severe clinical evolution) and Cluster B (severe clinical evolution). By this score, 22 patients (44%) presented a score below 14 points, while the remaining 28 patients (56%) scored 14 or more points and were assigned as Cluster B Ameratunga.

Grimbacher’s severity score applied to our CVID cohort disclosed a median of 5 points (range, 2 to 10 points). The cohort was divided according to the clustering algorithm using 5 points as cut-off, into Cluster A (non-severe clinical evolution) and Cluster B (severe clinical evolution). By this score, 12 patients (24%) presented a score below 5 points, while the remaining 38 patients (76%) scored 5 or more points and were assigned as Cluster B Grimbacher.

We then compared model fit by cross-tabulation of VISUAL with the frequency distribution of both severity scores using Fisher exact test and scatter plots. Correlation analysis was performed to compare performance between scores and analyzed by K-Means clustering algorithm (Supp. Fig. S2). A direct significant correlation was observed between VISUAL and Ameratunga’s severity score (p = 0.005). The increase in 1 unit (+) in VISUAL induced a positive correlation of severity score in 1.33-fold increase (95% CI 1.08; 1.57, p = 0.007). Regarding to correlation between VISUAL and Grimbacher’s severity score, a trend to positive correlation was observed between both tests (p = 0.19). Consecutively, we applied the logistic regression test taking 5 points as the cut-off in Grimbacher’s severity score. The increase in 1 unit (+) in VISUAL induced a positive correlation of severity score in 1.26-fold increase (95% CI 1.00; 1.58, p = 0.004).

**Figure 2:**
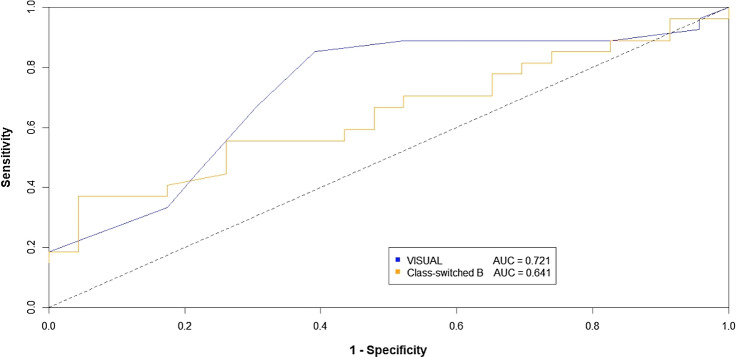
Receiver operating characteristic (ROC) curves for VISUAL versus isolated smB lymphocytes determination as a predictor of severity manifestations in CVID patients. Abbreviation: AUC, area under the curve. VISUAL showed significantly higher sensitivity (85% *vs* 55%, p = 0.01) and negative predictive value (77% *vs* 58%, respectively), while similar positive predictive value (72% *vs* 71%, respectively) and specificity (60% vs 73%, respectively) between both tests. The AUC obtained using the VISUAL score was superior to that derived using the isolated determination of smB lymphocytes, without statistical significance (AUC 0.721 *versus* 0.641).

The ROC analysis indicated a good performance of VISUAL for the discrimination between Cluster A and B of both severity scores. When we tested VISUAL performance in predicting severe clinical evolution (Cluster B for each clinical score) compared to that of the isolated determination of smB lymphocytes^4,40,41^, we found that VISUAL showed significantly higher sensitivity (85% *vs* 55%, p = 0.01) and negative predictive value (77% *vs* 58%, respectively), while similar positive predictive value (72% *vs* 71%, respectively) and specificity (60% vs 73%, respectively) between both tests. The AUC obtained using the VISUAL score was superior to that derived using the isolated determination of smB lymphocytes, without statistical significance (AUC 0.721 *versus* 0.641) (Fig. 2).

Both disparate clinical scores fitted with VISUAL in this real-life observational cohort. Ameratunga’s Cluster A (< 14 points) and Grimbacher’s Cluster A (< 5 points) showed markedly lower VISUAL values (median 9 points, IQR 4 and 8.5 points, IQR 3.5, respectively). This could be pointing to a milder form of CVID or to a less evolved disease. By contrast, Ameratunga’s Cluster B (≥ 14 points) Grimbacher’s Cluster B (≥ 5 points) showed high VISUAL values (median 11 points, IQR 3 and 11 points, IQR 3.25, respectively), better discriminating patients with severe clinical evolution. Not surprising, we evaluated time of evolution to Cluster B for each patient according to the clinical scores and did not observe statistically significant differences between clusters (data not shown).

Patients at diagnosis of CVID with VISUAL ≥ 10 (p30 of severity score) associated 8.94-fold higher odds of severe clinical prognosis with respect to VISUAL below this threshold (p = 0.001). In this study, Bootstrap 95% confidence intervals for the VISUAL score were as follows: sensitivity 85% (68–100%), specificity 60% (33–74%), and AUC of 0.721 (0.545 to 0.846).

**Figure 3:**
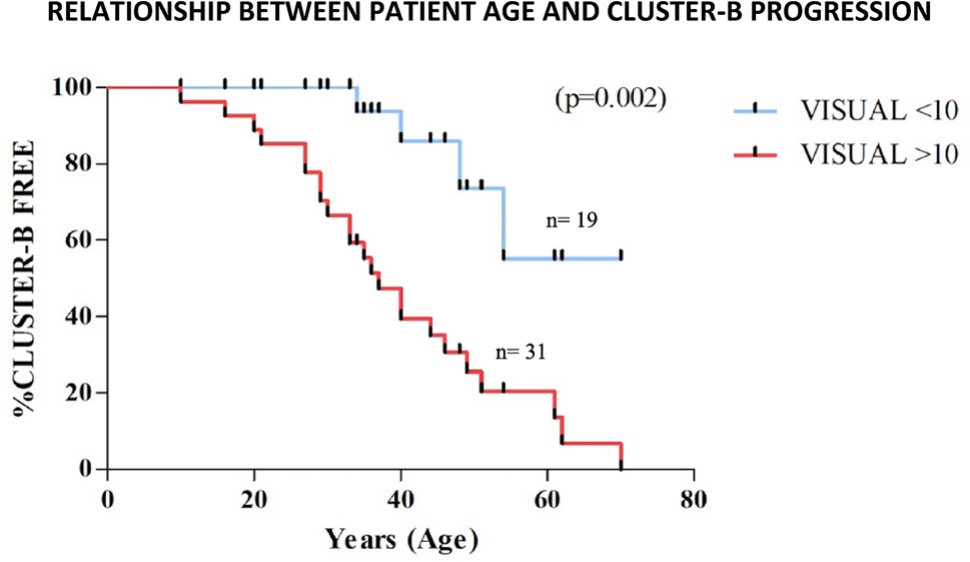
Relationship between patient age and development of complications (progression to Ameratunga’s Cluster B of severity) was assessed by Kaplan–Meier estimates for the VISUAL score. CVID patients with VISUAL ≥ 10 points progressed to cluster B faster than those with VISUAL < 10 (*p* = 0.0002).

The relationship between patient age and development of complications (progression to Cluster B of severity) was assessed by Kaplan–Meier estimates for the VISUAL score. CVID patients with VISUAL ≥ 10 points progressed to Cluster B faster than those with VISUAL < 10 (*P* = 0.0002) (Fig. 3), with a median age of 37-years-old *versus* 44 years-old in patients with VISUAL < 10 (n = 4). The mean time from age at CVID diagnosis to age at Cluster B-progression was 2 years (median 0, IQR 1).

## Discussion

The novel VISUAL score, using combined immunological biomarkers at CVID diagnosis, early predicted the severity of clinical manifestations or outcomes in our CVID cohort by two disparate CVID clinical scores, being independent of the course of the disease, with sensitivity of 85% and negative predictive value 77%. VISUAL showed superior sensitivity and accuracy to predict severity than the surrogate marker routinely used in clinical practice, namely smB phenotype alone. A cut-off of VISUAL 10 properly discriminated CVID patients with severe outcome (Cluster B). In our series, a large proportion of CVID patients showed severe evolution according to Ameratunga’s and Grimbacher’s clinical severity scores (56% and 76%, respectively), maybe reflecting a lack of referral of mild CVID cases in our health areas, which in turn may be associated with delay in diagnosis. Indeed, diagnostic delay may condition the development of complications derived from both the disease evolution and the recurrence of associated infections.

According to previous studies, only 15% of CVID patients can be attributed to a known genetic cause, presenting a relatively low strength of association to fully explain the inheritance pattern of CVID^42–44^. This could be explained either by a polygenic modes of inheritance and/or associated with epigenetic phenomena^45–48^. In fact, in most cases, the responsible gene or genes have not been identified. None of our patients could be excluded due to monogenic lesion.

VISUAL score is a non-invasive and easy-to-perform screening tool that would allow the identification of CVID patients at risk of presenting severe clinical evolution and that would benefit from close clinical follow-up and therapy. These results may support the hypothesis that the clinical manifestations and therefore the increased risk of complications in CVID patients is associated with deeper alteration of the analytical biomarkers early at diagnosis. smB lymphocytes are a predictor of inflammatory complications and outcomes in CVID, while do not predict infectious complications^4,32^. Nevertheless, more common infectious complications in CVID seem to be better predicted by functional tests of specific Ab production and by serum IgA^32,49^. Low serum total IgA levels has been associated with several CVID complications, as pneumonias and bronchiectasis^50^. High serum IgM levels associated with high smB cells has been linked to higher incidence of B-cell lymphoma’s in CVID^17,39^, while CD4^+^ T-lymphocytopenia may add on susceptibility risk of opportunistic infections in a subset of CVID patients. Studies such as the work of Mokhtari et al. suggest that certain laboratory factors might be related to developing severe complications in their cohort of 113 CVID patients^51^. In particular, high levels of IgM and alterations in CD4^+^ T-cells were described as factors associated with a more severe clinical phenotype and poorer survival^51–54^. Several studies have considered specific antibodies’ production within the assessment and especially in the IgRT decision for CVID patients^10^, which identify infectious risk mainly to encapsulated bacteria and respiratory viruses.

Besides, CD4^+^ T lymphocyte stages can guide clinicians on problematic complications, such as opportunistic and recurrent viral infections, gastrointestinal disease, lymphoma, autoimmunity and inflammation in CVID patients^33,55,56^. Although CVID diagnostic criteria exclude severe CD4^+^ T lymphocytopenia, the use of prolonged therapies such as corticosteroids is common as a first-line treatment for inflammatory manifestations of the disease, and may induce a temporary decrease of T-cells subpopulations as consequence^57,58^. Faced with a high clinical suspicion of CVID and marked CD4^+^ T lymphopenia, care must be taken when assessing these parameters that could be related to iatrogenesis. It is always necessary to exclude combined immune deficiency.

A major question is whether VISUAL might change over time, due to changes in CD4^+^ T lymphocytes numbers. Likewise, a genetic study based on an NGS panel of genes related to CVID and/or whole-exome sequencing might prove essential in the differential diagnosis and precision medicine approach. The integration of clinical, biological, immunological biomarkers, lifestyle and genetic defects conforms to the basis of desirable personalized healthcare, especially when a PIDD is suspected.

Recurrent respiratory infections and structural damage are factors associated with morbidity and mortality^59–61^. Bronchiectasis are considered a severity factor and classified as a moderate-severe complication depending on the extension of the lungs parenchyma^11,12^. In our cohort, 34.48% (10/29) of patients who had previous pneumonia developed bronchiectasis. It would be attractive to determine the role of bronchiectasis as a factor associated with disease progression. Nevertheless, due to the overlap of clinical phenotypes and the small sample size of our cohort, no relevant comparisons between a pure “infectious” versus “inflammatory” profile could be extracted.

Interestingly, up to 30% of our patients had moderate-severe infectious complications impacting their prognosis, which would have not been covered by smB lymphocytes phenotyping alone. This aspect highlights the necessity of combining markers to better define prognostic risk in CVID and the opportunity to avoid unfavorable evolution by using diagnostic and therapeutic strategies through early intervention. Thus, all 5 immunological variables scores integrate important information to CVID categorization. One of the weaknesses of this study is the relatively small size of the cohort and the retrospective design, necessary to develop the immunological-clinical fitness. However, due to the results obtained, it would be of interest to validate the VISUAL in a broader and prospective cohort of patients.

The proposed extended prognostic score proved to be a useful tool to classify CVID patients at diagnosis in order to anticipate and adjust follow-up and management. Notwithstanding that it is complicated to establish a linear distribution in all patients from a real-life scenario, so the performance of any instrument should be evaluated in individuals with quiescence or mild disease activity separately from patients with moderate to severe disease activity.

In conclusion, the VISUAL prognostic score might allow to predict and thus better establish a more appropriate follow-up requirement for each individual patient’s and to complement existing approaches forwards a more personalized care. Further studies are needed to prospectively validate VISUAL score.

## Supplementary Information

Supplementary Information.

## Abbreviations

CVID: Common variable immunodeficiency
IgRT: Immunoglobulin replacement therapy
PIDD: Primary immunodeficiency disorders
ROC: Receiver operating characteristic
smB: Switched memory B-lymphocytes
VISUAL: Variable immunodeficiency score by upfront analytical link
