# Supplementary material for: Variable immunodeficiency score upfront analytical link (VISUAL), a proposal for combined prognostic score at diagnosis of common variable immunodeficiency
Source: Sci Rep. 2021 Jun 9;11:12211. doi: 10.1038/s41598-021-91791-2 (PMC8190250; doi:10.1038/s41598-021-91791-2)
Supplement: Supplementary file 1 — Supplementary Information. [file 41598_2021_91791_MOESM1_ESM.docx]

**SUPPL TABLES. LEGENDS.**

**Suppl. Table 1.** Clinical manifestations of CVID patients. The median of evolution of the disease was 15.9 years (range, 1-31).

**Suppl. Table 2.** Description of known mutations in genetic studies of CVID patients.

**SUPPL. FIGURE LEGENDS.**

**Suppl. Fig. 1.** Density plots of: **A.** Ameratunga´s severity score *versus* VISUAL score, **B.** Grimbacher´s severity score *versus* VISUAL score.

**Suppl. Fig. 2.** Silhouette plots for the analysis of the two different clusters against VISUAL. Taking as reference both Ameratunga’s and Grimbacher’s severity score applied in our cohort. VISUAL presented a median of 10 points (range, 4 to 16), Ameratunga’s severity score disclosed a median of 14 points (range, 8 to 21) and Grimbacher’s severity score with a median of 5 points (range, 2 to 10). Patients with severe complications or more than one moderate complication amounted to 14 or more score points in Ameratunga’s severity score and to 3 or more score points in Grimbacher’s severity score. The cohort was divided according to the clustering algorithm using the medians of both severity scores, into Cluster A (no severe clinical evolution) and Cluster B (severe clinical evolution). Created with BioRender.com.

**SUPPL. TABLES.**

**Suppl. Table 1.**

| Patients | Clinical Manifestation |
| --- | --- |
| 1 | Asymptomatic splenomegaly, mild bronchiectasis, mild lymphadenopathy, acute sinusitis, uncomplicated pneumonia |
| 2 | Severe infectious enteritis, acute sinusitis, moderate asthma. |
| 3 | Complicated pneumonia, mild bronchiectasis, asymptomatic splenomegaly, mild asymptomatic cytopenia, Giardia, acute sinusitis |
| 4 | Uncomplicated pneumonia, mild bronchiectasis, symptomatic splenomegaly, otitis externa, mild asymptomatic cytopenias. |
| 5 | Uncomplicated vitamin deficiency associated with Intestinal Parasitosis (Oxyuriasis), mild asymptomatic cytopenias, uncomplicated pneumonia, mild bronchiectasis, acute sinusitis. |
| 6 | Cytopenia requiring treatment, complicated pneumonia, uncomplicated shingles, asymptomatic splenomegaly, acute sinusitis. |
| 7 | Lung surgery (left-lower lobectomy), asymptomatic splenomegaly, uncomplicated pernicious anemia, mild lymphadenopathy. |
| 8 | Severe eczema, symptomatic splenomegaly, mild lymphadenopathy, mild asymptomatic cytopenia. |
| 9 | Food and antibiotic allergies, complicated pneumonia, chronic rhinosinusitis, asymptomatic splenomegaly, Helicobacter pylori responding to treatment. |
| 10 | Non-life threatening abscesses, autoimmune gastritis, complicated pneumonia, autoimmune thyroiditis |
| 11 | Mild bronchiectasis, complicated pneumonia, asymptomatic splenomegaly, chronic rhinosinusitis. |
| 12 | Complicated pneumonia, non-life threatening abscesses, mild eczema, uncomplicated shingles. |
| 13 | Complicated pneumonia, sinusitis, mild bronchiectasis, Helicobacter pylori responding to treatment, symptomatic increase in liver enzymes, external otitis. |
| 14 | Malignancy (recurrent non-Hodgkin lymphoma plus Hodgkin lymphoma) |
| 15 | Splenectomy (due to idiopathic thrombocytopenic purpura), acute sinusitis, arthralgia, asymptomatic increase in liver enzymes. |
| 16 | Arthritis, enteropathy celiac disease-like, splenectomy, acute sinusitis, mild eczema. |
| 17 | Pericarditis, chronic rhinosinusitis, mild asthma, uncomplicated pneumonia, asymptomatic splenomegaly. |
| 18 | Severe GLILD, viral hepatitis responding to treatment, symptomatic splenomegaly, mild asymptomatic cytopenia |
| 19 | Mild inflammatory bowel disease, Addison´s disease, otitis media, uncomplicated pneumonia, asymptomatic splenomegaly, mild asymptomatic cytopenias. |
| 20 | Reaction to IVIG, cytopenias requiring treatment, asymptomatic splenomegaly, otitis media, uncomplicated UTI´s, uncomplicated pneumonia. |
| 21 | Addison´s disease, multiple antibiotic allergies, osteomyelitis, asymptomatic splenomegaly. |
| 22 | Severe SLE, cytopenias requiring treatment, Giardia, uncomplicated UTI´s, arthralgia |
| 23 | Meningitis with no sequelae, symptomatic splenomegaly, oral ulceration, mild lymphadenopathy, mild asthma, acute sinusitis. |
| 24 | Splenectomy (due to idiopathic thrombocytopenic purpura), acute sinusitis, rhinitis. |
| 25 | Mild inflammatory bowel disease responding to budesonide, asymptomatic increase in liver enzymes, asymptomatic splenomegaly, mild asymptomatic cytopenias. |
| 26 | Symptomatic splenomegaly, cytopenias requiring treatment, uncomplicated pneumonia. |
| 27 | Primary biliary cirrhosis with liver transplantation, cytopenias requiring treatment, asymptomatic splenomegaly, uncomplicated pneumonia. |
| 28 | Malignancy (non-Hodgkin lymphoma), uveitis responding to treatment, uncomplicated pneumonia, arthralgia. |
| 29 | Malignancy (gastric cancer), cytopenias requiring treatment, uncomplicated pneumonia. |
| 30 | Uncomplicated pneumonia, acute sinusitis, uncomplicated EBV viremia. |
| 31 | Uncomplicated singles, chronic rhinosinusitis, arthralgia, mild asymptomatic cytopenia, mild lymphadenopathy. |
| 32 | Extensive incl. Sarcoid-like granulomatous disorder, splenectomy (due to symptomatic splenomegaly) |
| 33 | Granulomatous involvement of urinary tract, lichen planus, type 1 diabetes, uncomplicated pneumonia |
| 34 | Poorly responsive treatment cytopenia required Rituximab, asymptomatic splenomegaly, Giardia. |
| 35 | Severe pulmonary dysfunction with extensive bronchiectasis, complicated pneumonia |
| 36 | Extensive incl. Sarcoid-like granulomatous disorder, cholecystitis, uncomplicated vitamin deficiency. |
| 37 | Poorly responsive treatment cytopenia required Rituximab, asymptomatic splenomegaly, uncomplicated pneumonia, acute sinusitis. |
| 38 | Extensive incl. Sarcoid-like granulomatous disorder, Giardia, asymptomatic increase in liver enzymes. |
| 39 | Severe malabsorption protein-losing enteropathy, cytopenias requiring treatment, Giardia, mild lymphadenopathy. |
| 40 | Malignancy (non-Hodgkin lymphoma), connective tissue disorder, uncomplicated pneumonia |
| 41 | Complicated pneumonia, mild bronchiectasis, chronic rhinosinusitis. |
| 42 | Poorly responsive treatment cytopenia required Rituximab, asymptomatic splenomegaly, uncomplicated pneumonia, acute sinusitis. |
| 43 | Mild cellulitis, otitis media, uncomplicated pneumonia. |
| 44 | Cytopenias requiring treatment, symptomatic splenomegaly, Helicobacter pylori responding to treatment, uncomplicated vitamin deficiency, uncomplicated CMV viremia, otitis externa. |
| 45 | Sjogren’s syndrome, rhinitis, asthma, complications from long-term steroids, mild lymphadenopathy. |
| 46 | Cytopenias requiring treatment, multiple allergies antibiotics and reactions to IVIG, complicated pneumonia. |
| 47 | Viral meningitis with no sequelae, chronic rhinosinusitis. |
| 48 | Uncomplicated pneumonia, mild bronchiectasis, Severe cutaneous lupus, Malignancy (non-Hodgkin lymphoma). |
| 49 | Complicated pneumonia, mild bronchiectasis, reactions to IVIG. |
| 50 | Viral hepatitis responding treatment, cutaneous vasculitis, cytopenias requiring treatment, complicated pneumonia. |

**Suppl. Table 2.**

| Patients | Genetic Study |
| --- | --- |
| Patient 1 | Mutation in PLCG2 and LRBA (both in heterozygosis). |
| Patient 2. | Mutations in VPS13B, AIRE, NLRP12, CHD7, NCF2 (all in heterozygosis). |
| Patients 3 and 4 (consanguineous patients) | Mutation c.310 T>C (p.Cys104Arg) and c.512 T > G (p.Leu171Arg in heterozygosis of the TNFRSF13B gene. |
| Patient 5. | Homozygous mutations in PLCG2 and TACI (TNFRSF13B), and heterozygous mutation in LRBA. |

**SUPPL. FIGURE.**

**Suppl. Fig. 1.**


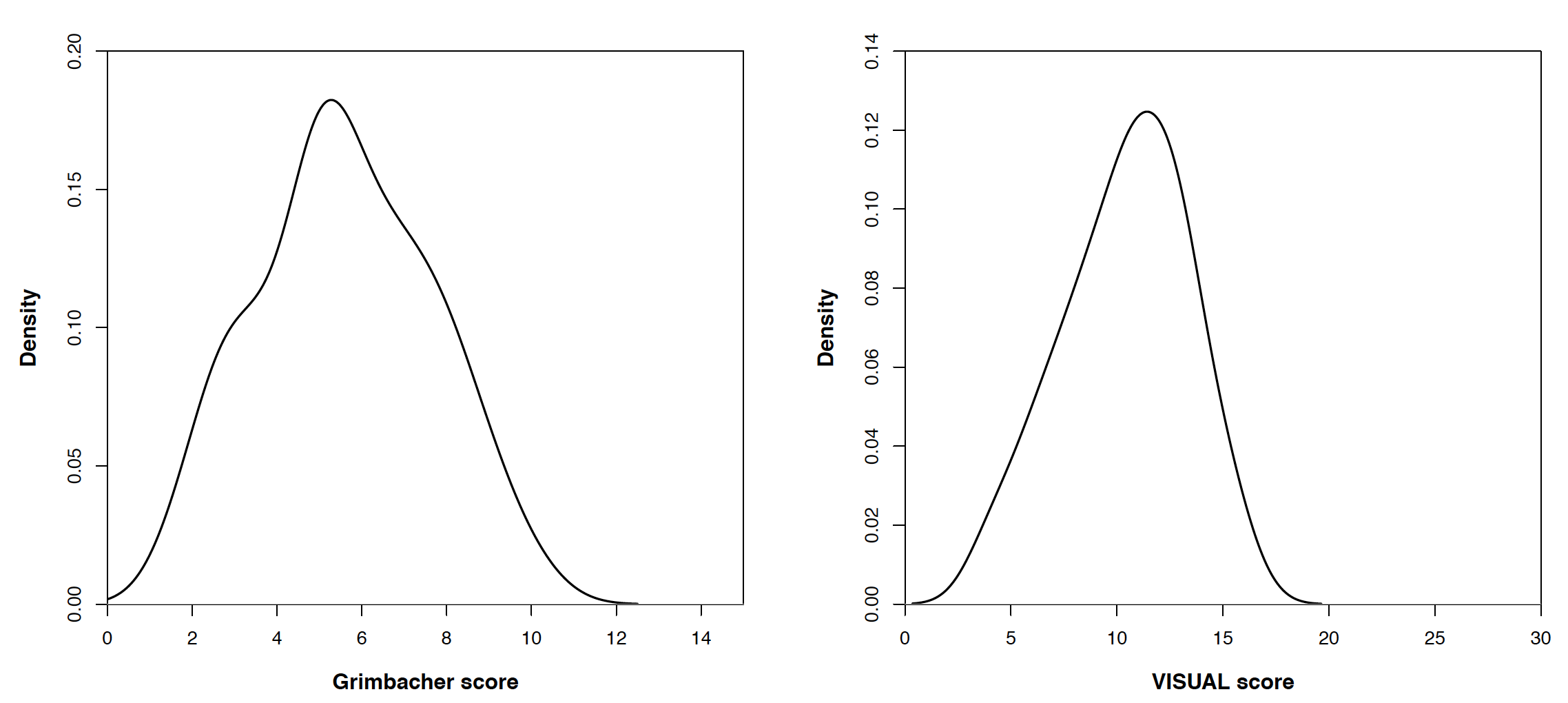


A

B

median 14 pts.

median 10 pts.

median 10 pts.

median 5 pts.

**Suppl. Fig. 2.**

**
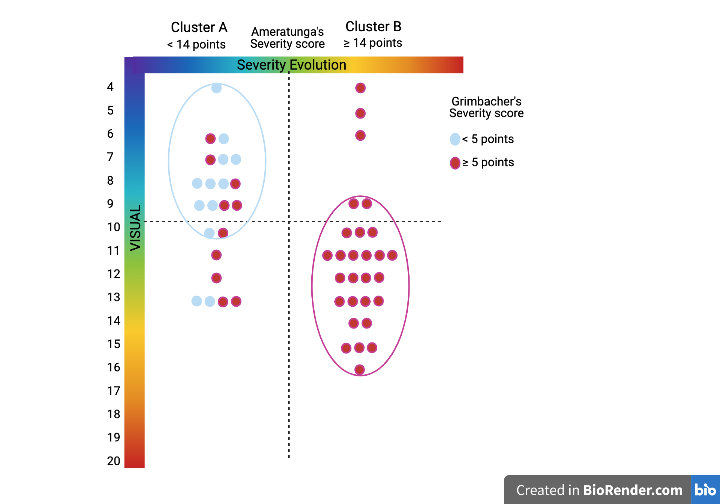
**
